# 2024 Healthcare Delivery Science: Innovation and Partnerships for Health Equity Research (DESCIPHER) Symposium

**DOI:** 10.1002/lrh2.70042

**Published:** 2025-12-04

**Authors:** Amytis Towfighi, Allison Z. Orechwa

**Affiliations:** ^1^ Southern California Healthcare Delivery Science Center California Los Angeles USA; ^2^ Southern California Clinical and Translational Science Institute California Los Angeles USA; ^3^ Los Angeles County Department of Health Services California Los Angeles USA; ^4^ Department of Neurology University of Southern California Keck School of Medicine California Los Angeles USA; ^5^ Los Angeles General Medical Center California Los Angeles USA

**Keywords:** conference, engagement, healthcare delivery science, lived experience, symposium

## Abstract

**Background:**

The Southern California Healthcare Delivery Science Center organizes an annual symposium for a broad audience interested in health innovation.

**Aims:**

The 2024 symposium convened healthcare professionals, researchers, policymakers, and advocates to explore innovative strategies for advancing health equity.

**Materials & Methods:**

Organizers assembled panels of patients, health system leaders, and researchers to present their perspectives on four primary themes: (1) conceptual frameworks for social determinants of health, (2) community‐engaged healthcare delivery interventions, (3) healthcare system‐based strategies and innovations, and (4) the ethical use of emerging technologies. The agenda also included poster presentations and interactive break‐out sessions.

**Results:**

Twenty presenters and facilitators engaged attendees in discussions throughout the day‐long symposium. A common theme was understanding social determinants as fundamental, intermediate, and proximate drivers of health inequities. Strategies to bridge these gaps included interdisciplinary collaboration, engaging individuals with lived experience, healthcare system‐based and community‐centered interventions, ethical use of artificial intelligence, and policy reform.

**Discussion:**

Presentations emphasized the importance of interdisciplinary collaboration, innovation, and policy reform in addressing social determinants of health and achieving equity. They also highlighted the significance of lived experience, community involvement, and data‐driven strategies in advancing healthcare delivery science.

**Conclusion:**

The 2024 Healthcare Delivery Science Symposium successfully convened broad stakeholders to exchange ideas and proven strategies for advancing health equity.

## 1 Introduction

**FIGURE 1:**
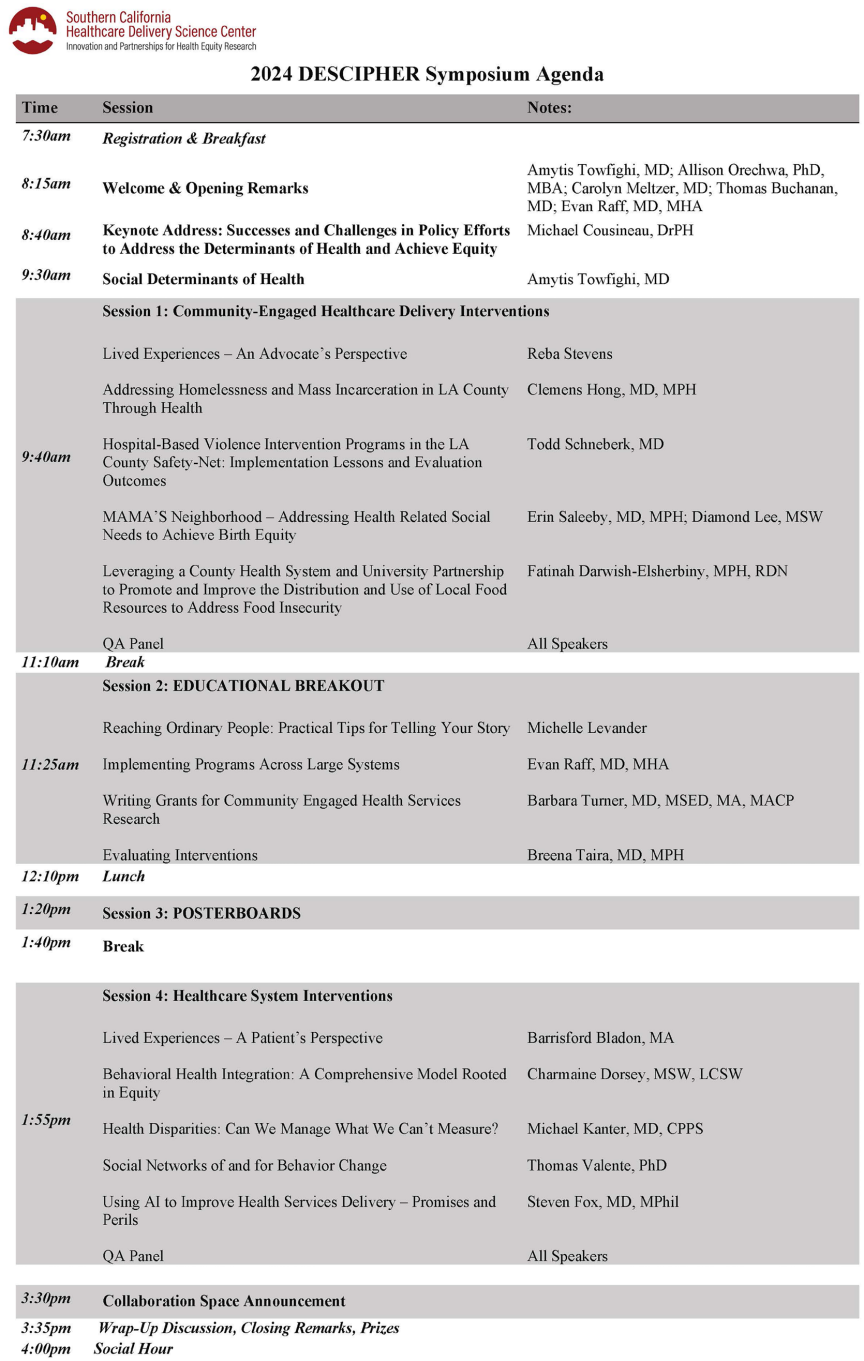
Symposium agenda.

Held in October of 2024, the annual Healthcare Delivery Science: Innovation and Partnerships for Health Equity Research (DESCIPHER) Symposium convened healthcare professionals, researchers, policymakers, and advocates to explore innovative strategies for advancing health equity (Figure 1). Organized by the Southern California Healthcare Delivery Science (HDS) Center in collaboration with the Keck School of Medicine at USC and the Los Angeles County Department of Health Services, the symposium highlighted the importance of addressing medical and social determinants of health (SDOH). This article summarizes key presentations, discussions, and themes. Recordings and slides are available on the Center website: https://sites.usc.edu/hdscenter/2024‐symposium‐recordings/.

## 2 Welcome Remarks

The day‐long symposium opened with a welcome from HDS Center leaders, Amytis Towfighi, MD, and Allison Orechwa, PhD, followed by brief remarks by key supporters. Carolyn Meltzer, MD, Dean of the Keck School of Medicine of the University of Southern California, and Thomas A. Buchanan, MD, Director of the Southern California Clinical and Translational Science Institute, underscored the importance of interdisciplinary collaboration and the Center's role in bridging healthcare gaps. Evan Raff, MD, MHA, Director of Specialty Care for Los Angeles County Department of Health Services (DHS), presented DHS's strategy to patient‐centered care delivery, which integrates elements of health equity, workflow innovation, and care coordination to improve outcomes.

## 3 Keynote Address

The keynote address, delivered by Michael Cousineau, DrPH, focused on the successes and challenges in policy efforts aimed at addressing SDOH. Cousineau provided a historical overview of healthcare policies, highlighting notable milestones and obstacles. He argued that, despite high healthcare spending, the United States has poor health outcomes. He explored the fragmented nature of health policies and proposed reforms that could lead to health equity, with California's efforts toward universal coverage cited as a potential model for addressing social determinants on a broader scale. He called on researchers to “develop and support new and modern notions of healthcare systems” through rigorous studies that are multidisciplinary, patient‐focused, and policy‐relevant.

## 4 Lectures and Breakout Sessions

The symposium was organized around four primary themes: (1) conceptual frameworks for SDOH; (2) community‐engaged healthcare delivery interventions; (3) healthcare system‐based strategies and innovations, including behavioral health and health disparity measurement; and (4) the ethical use of emerging technologies, such as social network analysis and artificial intelligence (AI), in healthcare delivery. These sessions provided a comprehensive platform for in‐depth discussions, uniting diverse voices from healthcare, research, policy, and the community to foster innovative strategies for advancing health equity and improving overall population health outcomes.

## 5 Conceptual Framework for Social Determinants of Health

Amytis Towfighi, MD, Director of the HDS Center, presented models for understanding SDOH from the World Health Organization, the Bay Area Regional Health Inequities Initiative, and the NINDS Social Determinants of Health Framework [1, 2, 3]. Drawing from her experiences in Kenya and the U.S., Towfighi explained how policies and environmental factors, such as access to clean water and healthcare, impact health outcomes. She emphasized that it is critical to use conceptual frameworks to better understand the interplay between social determinants and to visualize entry points for intervention. She encouraged attendees to consider these frameworks in the day's discussions, with a particular focus on cross‐sector collaborations.

## 6 Community‐Engaged Healthcare Delivery Interventions

This session spotlighted innovative programs that integrate community engagement to address health inequities in Los Angeles.

### 6.1 Lived Experiences—The Advocate's Perspective

Reba Stevens, a seasoned community advocate, shared her personal journey of resilience and her experience spending 20 years unhoused in Los Angeles. She discussed the deep‐rooted issues that often go unrecognized and unaddressed. She emphasized that the lack of stable housing, compounded by adverse social conditions and limited healthcare access, can lead individuals down paths they are often unable to articulate or control. Stevens highlighted the critical role of involving people with lived experiences, as they can help healthcare professionals gain insight into the challenges faced by vulnerable populations. Drawing from her own life, she advocated for a more empathetic approach in healthcare that considers the unique experiences of individuals. She remarked, “It's really the job of people who are in these spaces to get to know who people really are. We are not asking people about the root causes of whatever it is that they've experienced. And the beauty of having those conversations is that it affords…the person you're talking to [the ability] to reach back inside and touch those things, knowing that on the other side there's a hand to assist and serve…If we don't know the truth about what is really going on, then there is no solution.”

### 6.2 Addressing Homelessness and Mass Incarceration in LA County

Clemens Hong, MD, MPH, Director of LA County's Whole Person Care program, discussed a holistic approach to addressing homelessness and the effects of mass incarceration on health. He outlined the program's efforts to engage with communities directly and to involve individuals with lived experience in program design and delivery. This unique initiative focuses on investing in local communities by creating job opportunities and bridging healthcare gaps for marginalized populations. Impact metrics from 2023 include helping 432 households in the Homeless Prevention Unit and serving 18,542 people with Permanent Supportive Housing, with 86% and 85% retaining housing, respectively.

Hong also emphasized the importance of working with people who have firsthand experience, as they bring invaluable insights into effective intervention strategies. “Without engagement, you can't do anything. It's the foundation for everything. The key to that engagement is building trust, and you build trust through love… I will tell you that when I go out to the communities and I visit our Community Programs teams, when I visit with the CBOs that are delivering this care, I see it everywhere. These are folks that take the extra hour, that take the extra time to…deliver love. And they build that engagement…that's the entry point into care delivery in this space…This is what makes…our community programs successful…our partners, the people with lived experience, the people that are on the streets delivering this mission‐oriented work every single day.”

### 6.3 Hospital‐Based Violence Intervention Programs

Todd Schneberk, MD, presented on hospital‐based violence intervention programs within the Los Angeles County DHS system that serve victims of interpersonal violence in partnership with community‐based organizations. The programs deploy individuals with lived experience to connect with victims at the bedside, providing a bridge to critical support services. One community collaborator described these individuals as a critical facilitator because participants think, “Oh, they're from my community. They know the crossroads. They know what's happening in the communities.” He highlighted the cycle of trauma that often brings individuals back into dangerous situations and stressed the need for wraparound care that extends beyond immediate physical health needs. Schneberk shared preliminary data from cost–benefit analyses, which reveal that early intervention and social support can reduce recurrent violence and ultimately improve both individual and community outcomes while reducing overall cost.

### 6.4 MAMA'S Neighborhood—Addressing Birth Equity

Erin Saleeby, MD, MPH and Diamond Lee, MSW presented MAMA's Neighborhood [4], a program designed to address health‐related social needs and improve birth equity outcomes. The initiative tackles inequities in maternal and infant health, particularly within Black and other underserved communities, which have elevated risk of adverse social determinants such as substance use, intimate partner violence, and depression. Saleeby and Lee discussed the program's unique combination of components, including stress assessments, team‐based care, social services coordination, and expanded mental health services for new mothers, in addition to consultants with lived experience. This approach, which has led to significantly lower rates of preterm births (18%–10% over the course of implementation), not only provides much‐needed resources but also empowers individuals to advocate for themselves within the healthcare system.

### 6.5 Leveraging Partnerships to Address Food Insecurity

Fatinah Darwish‐Elsherbiny, MPH, RDN, highlighted a collaboration between Los Angeles County's Department of Public Health, DHS, and local universities to combat food insecurity, a key factor that heightens cardiovascular risk. The learning collaborative focuses on systematically expanding access to nutritious food through partnerships with grocery stores, food rescue programs, and community organizations. Patients are screened for food insecurity during healthcare visits and connected to food resources and nutrition education [5]. Ms. Darwish‐Elsherbiny underscored that such partnerships can play a critical role in addressing diet‐related health issues, fostering sustainable change in health outcomes for vulnerable populations.

## 7 Educational Breakout Sessions

Attendees participated in breakout sessions covering practical topics: storytelling for health impact (Michelle Levander), implementing programs at scale (Evan Raff, MD, MHA), grant writing for community health research (Barbara Turner, MD, MSED, MA, MACP), and evaluating interventions (Breena Taira, MD, MPH). These sessions provided tools and strategies for effectively engaging communities and measuring the impact of health programs.

## 8 Healthcare System Interventions

This session explored innovative, system‐based strategies for addressing SDOH and fostering equitable, effective healthcare.

### 8.1 Lived Experience—The Patient Perspective

Barrisford Bladon, MA, a stroke survivor and occupational therapist, shared his powerful story of resilience and transformation. Following a transient ischemic attack (TIA) and subsequent stroke, Bladon faced significant barriers during his recovery journey, from limited resources to healthcare system gaps. He said, “I spent 7 days in the ICU, and to put salt on that wound the doctor told me everything that he didn't think I would be able to do again. I'm a strong man of faith, so that combined with his words lit a fire under me that I can't describe.” Bladon used this as motivation to recover, return to school, and pursue a career in occupational therapy, saying, “I had to become educated for doctors to respect me.” He emphasized advocacy for both patients and providers, encouraging patients to assert their needs and healthcare teams to understand the importance of empathy and individualized support. “[patients] don't want a Bandaid, they want real solutions, they want you to partner with them.” He shared the motto he tells all his clients: “Don't let anyone tell you what you can't do. You are not disabled. You are differently abled. And with motivation and rehabilitation you can achieve the goals you set for yourself.”

### 8.2 Behavioral Health Integration

Charmaine Dorsey, MSW, LCSW, presented the DHS Behavioral Health Integrated Model within primary care, designed to address patients' social and emotional needs alongside their physical health [6]. The model embeds standardized social needs screening tools within electronic medical records. Once needs are identified, a team of licensed clinical social workers, medical case workers, substance use disorder counselors, and community health workers collaborates with primary care providers—using trauma‐informed care principles—to connect patients with resources. By integrating behavioral health services, this team‐based care model strengthens clinical decision‐making, reduces stigma, and ensures patients feel genuinely heard, supported, and valued within the healthcare system.

## 9 Health Inequities: Can we Manage What we Don't Measure?

Michael Kanter, MD, CPPS, tackled the critical question of measuring health inequities, focusing on the adage “you can't manage what you don't measure.” Kanter emphasized that a robust framework for measuring inequities is essential for advancing quality improvement, research, and accountability in healthcare. He described a language concordance initiative at Kaiser Permanente that increased the number of physicians fluent in their patients' languages through monetary incentives and educational opportunities. The team measured blood pressure, colon cancer screening rates, and other quality measures and observed better rates in language concordant versus non‐concordant patient‐physician pairs. He highlighted that “public reporting and transparency of disparity data have potential to be helpful and allow communities and delivery systems to work together more strategically.”

## 10 Social Networks of and for Behavior Change

Thomas Valente, PhD, discussed the impact of social networks in influencing behavior change, exploring social network analysis as a tool for understanding relationship dynamics within various populations. His talk highlighted how social networks can affect behaviors, from adolescent substance abuse to decision‐making among healthcare providers. By identifying central figures within networks—those who act as influencers or opinion leaders—healthcare interventions can be structured to harness these relationships and drive positive behavioral shifts. Valente emphasized that social network analysis can guide the design of effective interventions, supporting healthcare providers in implementing targeted, community‐based solutions.

## 11 Using AI to Improve Health Services Delivery

Steven Fox, MD, MPhil, examined the role of AI in healthcare, particularly its promises and limitations in diagnostic and treatment planning. He outlined how AI, while a powerful tool for tasks like image interpretation and early diagnosis, requires careful oversight due to the potential for bias in data and lack of interpretability in decision‐making. Fox used the analogy of self‐driving cars to explain that while AI can make processes more efficient, healthcare cannot tolerate the same level of risk associated with non‐human oversight. He warned of the dangers of over‐reliance on AI without proper human guidance, advocating for its use as an aid to, rather than a replacement for, human judgment. Fox concluded that AI's most significant potential lies in enhancing, not substituting, the clinician's role, particularly in patient‐centered care.

## 12 Conclusion

The 2024 Healthcare Delivery Science Symposium emphasized the importance of interdisciplinary collaboration, innovation, and policy reform in addressing social determinants of health and achieving equity. The discussions highlighted the significance of lived experience, community involvement, and data‐driven strategies in advancing healthcare delivery science. The symposium concluded with a call to action for continued partnerships and a commitment to driving healthcare delivery transformation for all.

## Conflicts of Interest

The authors declare no conflicts of interest.
